# Changes in the neurochemistry of athletes with repetitive brain trauma: preliminary results using localized correlated spectroscopy

**DOI:** 10.1186/s13195-015-0094-5

**Published:** 2015-03-15

**Authors:** Alexander P Lin, Saadallah Ramadan, Robert A Stern, Hayden C Box, Christopher J Nowinski, Brian D Ross, Carolyn E Mountford

**Affiliations:** Center for Clinical Spectroscopy, Department of Radiology, Brigham & Women’s Hospital, Harvard Medical School, 4 Blackfan Street HIM-820, Boston, MA 02115 USA; Centre for MR in Health, School of Health Sciences, University of Newcastle, Newcastle, NSW 2308 Australia; Center for the Study of Traumatic Encephalopathy, Boston University School of Medicine, Boston, MA 02118 USA; BU Alzheimer’s Disease Center, Boston University School of Medicine, Boston, MA 02118 USA; Clinical Spectroscopy, Huntington Medical Research Institutes, Pasadena, CA 91105 USA; Sports Legacy Institute, Waltham, MA 02451 USA

## Abstract

**Introduction:**

The goal was to identify which neurochemicals differ in professional athletes with repetitive brain trauma (RBT) when compared to healthy controls using a relatively new technology, *in vivo* Localized COrrelated SpectroscopY (L-COSY).

**Methods:**

To achieve this, L-COSY was used to examine five former professional male athletes with 11 to 28 years of exposure to contact sports. Each athlete who had had multiple symptomatic concussions and repetitive sub concussive trauma during their career was assessed by an experienced neuropsychologist. All athletes had clinical symptoms including headaches, memory loss, confusion, impaired judgment, impulse control problems, aggression, and depression. Five healthy men, age and weight matched to the athlete cohort and with no history of brain trauma, were recruited as controls. Data were collected from the posterior cingulate gyrus using a 3 T clinical magnetic resonance scanner equipped with a 32 channel head coil.

**Results:**

The variation of the method was calculated by repeated examination of a healthy control and phantom and found to be 10% and 5%, respectively, or less. The L-COSY measured large and statistically significant differences (*P ≤*0.05), between healthy controls and those athletes with RBT. Men with RBT showed higher levels of glutamine/glutamate (31%), choline (65%), fucosylated molecules (60%) and phenylalanine (46%). The results were evaluated and the sample size of five found to achieve a significance level *P =* 0.05 and a power of 90%. Differences in N-acetyl aspartate and myo-inositol between RBT and controls were small and were not statistically significance.

**Conclusions:**

A study of a small cohort of professional athletes, with a history of RBT and symptoms of chronic traumatic encephalopathy when compared with healthy controls using 2D L-COSY, showed elevations in brain glutamate/glutamine and choline as recorded previously for early traumatic brain injury. For the first time increases in phenylalanine and fucose are recorded in the brains of athletes with RBT. Larger studies utilizing the L-COSY method may offer an in-life method of diagnosis and personalized approach for monitoring the acute effects of mild traumatic brain injury and the chronic effects of RBT.

## Introduction

There is increasing recognition of long-term neurological difficulties from head injuries, especially from repetitive concussive and sub-concussive brain trauma. Chronic traumatic encephalopathy (CTE) is a progressive neurodegenerative disease, found post mortem, in individuals with histories of repetitive brain trauma (RBT), such as contact sport athletes and combat military personnel [1]. Early symptoms of RBT include impaired memory and executive functioning. Mood disturbance, including depression, apathy, suicidal tendencies and behavior change, follow as do impulsivity and aggression [1]. Later in the disease course, dementia can develop as well as motor disturbance in some people [2]. Autopsy studies have revealed a distinct pattern of neuropathological changes known as CTE which includes: tau-immunoreactive proteins in the cerebral cortex; neurofibrillary and glial tangles; generalized atrophy; enlarged ventricles; degeneration of white matter fiber bundles; cavum septum pellucidum; and a relative absence of beta-amyloid (Aβ) deposits [3,4].

The neuropathological diagnosis of CTE is well documented but there is a need for an *in vivo* diagnosis to provide an in life and personalized approach to manage this disease. An in life test will also facilitate research into the risk factors, epidemiology, prevention and treatment. Magnetic resonance spectroscopy (MRS) can be used to noninvasively record neurochemical changes as a result of RBT. One-dimensional MRS allows neurochemicals, mobile on the MR timescale, to be monitored noninvasively in a clinical MR scanner [5]. However, many of these neurochemicals overlap in the one-dimensional MR spectrum making it difficult to determine the precise level of change to each chemical population. A single chemical species can be measured at one time using MR spectral editing techniques [6-8] but these do not allow comparison of how the neurochemicals alter in relation to one another [9,10].

The introduction of Localized (L) COrrelated SpectroscopY (L-COSY) overcame this issue [11,12]. L-COSY utilizes a simple three-pulse sequence:

$$ 9{0}_{\phi 1}-18{0}_{\phi 2}-\mathrm{t}1-9{0}_{\phi 3}-{\mathrm{Acq}}_{\phi 4}\left(\phi \kern0.5em \mathrm{is}\ \mathrm{phase}\right) $$

whereby each row in the final spectrum is acquired in succession and t1 time delay is incremented by a set value, thereby providing a two-dimensional spectrum. In an L-COSY spectrum, a ‘cross peak’ off the diagonal indicates scalar coupling between the two protons it connects on the diagonal, thus providing chemical specificity not available to conventional one-dimensional MRS. Using data collected in a whole body commercially available scanner at 3 T, there are many more molecules now open for inspection in the brain using the L-COSY method with higher certainty. This L-COSY method has been applied successfully to other organs including muscle and bone marrow [13] and brain [11]. The advantage of L-COSY is that it allows for unambiguous identification and measurement of metabolites, lipids and macromolecules, compared to the one-dimensional MR spectra [14]. For the brain, the L-COSY allows over 35 different resonances to be inspected, each of which can be related to function, disease or injury [11].

The L-COSY protocol, using the 32 channel head coil, was previously reported to be effective for studying the human brain with a signal to noise ratio sufficient to monitor the neurochemicals from healthy brain and glioma [11]. The spectral quality allowed similar chemical information, recorded from cultured cells at 8.4 T, to be seen *in vivo* from the brain of patients with glioblastoma in a 3 T clinical scanner. In the glioma study, the changes to neurochemistry were large. Much smaller changes were anticipated from subjects with RBT. Here, we report the results of a pilot study, using the L-COSY method, on chronic sports-induced repetitive head injury in elite athletes compared with healthy age-matched controls with no history of head injury. Although the cohort is small, we report large differences in neurochemistry, with statistical significance, not previously described for RBT.

## Methods

### Magnetic resonance

#### Hardware

Data were acquired on a 70 cm wide bore Siemens Verio (Siemens AG, Erlangen, Germany) using the operating software VB17 using the 32 channel head coil (Siemens AG).

### MRI and MRS protocol

Prior to the spectroscopy data collection, routine brain magnetic resonance imaging (MRI) was performed with axial 3D-MPRAGE and reconstructed in the sagittal and coronal planes with 2 mm slice resolution for accurate localization of the voxel. The posterior cingulate gyrus (PCG), predominantly comprising gray matter, was chosen for examination as it had been reported to be sensitive to traumatic brain injury [15,16]. Furthermore, post mortem CTE studies show deposition of tau protein in the cortical gray matter areas including the cingulate [3].

The L-COSY protocol was undertaken as described in Ramadan *et al*. [11] with a 32 channel head coil, using 64 increments with 8 averages, and a repetition time (TR) of 1.5 seconds resulting in an acquisition time of 12.8 minutes. The data were acquired from a voxel in the PCG (size 3 × 3 × 3 cm^3^), acquired vector size 1,024 points; acquisition time 512 ms; spectral width in F2 2,000 Hz and spectral width in F1 1,250 Hz (0.8 ms increment size).

Localized shimming was undertaken by adjustment of zero- and first-order shim gradients using the automatic B0 field mapping technique supplied by the vendor (Siemens AG) followed by manual adjustment of zero-order shim gradients to achieve a resulting peak width of water at half-maximum that was 14 Hz or less. Following frequency adjustment, water-selective suppression was achieved using the WET-technique [17] programmed into the pulse sequence.

### Subjects

The study was approved by the local institutional review boards (Partners Human Research Committee and Boston University Medical Campus Institutional Review Board) and was compliant with the Health Insurance Portability and Accountability Act. All subjects provided informed consent and consent for publication.

Five retired professional male athletes participated in the study (43.6 ± 10.8 years) (Table 1). They had 11 to 28 years (17.4 ± 7.2 years) of exposure to repetitive brain trauma in contact sports, including American football (n = 3), professional wrestling (n = 1) and baseball (n = 1). Each athlete had experienced multiple symptomatic concussions (including several concussions with loss of consciousness) as well as repetitive sub-concussive trauma during his career. The subjects were all examined at the chronic stage of injury 3 to 25 (13.8 ± 10.1) years since the end of their careers and had no recently reported head injuries. Subjects were each evaluated by an experienced neuropsychologist (RAS) within a few days of MR examination via a personal interview; a semi quantitative neuropsychological test score was assigned to each (see Table 1). All five former athletes had reported clinical symptoms associated with either prolonged post-concussive syndrome or early CTE, including headaches, memory loss, confusion, impaired judgment, impulse control problems, aggression and depression [2]. Five healthy men, who were not professional athletes, were recruited as control subjects. They were age and weight matched to the athletes (45.2 ± 12.6 years; no significant difference from subjects), with no history of brain trauma.

**Table 1:** **Description of RBT cohort**

**Case**	**Age group**	**Sport**	**Professional years**	**Total years**	**Cognitive and neuropsychiatric symptom severity**
**A**	30 to 35	Football/Wrestling	3	11	+
**B**	30 to 35	Football	7	28	++
**C**	30 to 35	Baseball^a^	7	11	++
**D**	40 to 55	Football	9	21	++
**E**	40 to 55	Football	7	16	+++

^a^In addition to baseball-related concussions, subject also had several concussions from non-sports-related activities.

+mild cognitive and neuropsychiatric symptoms; ++moderate cognitive and neuropsychiatric symptoms; +++significant cognitive and neuropsychiatric symptoms. RBT, repetitive brain trauma.

To determine the variation of each cross peak using this L-COSY sequence, data were accrued four times in succession from phantom solutions of the major metabolites (N-acetyl aspartate, creatine, choline and myo-inositol) at physiological concentrations and pH as well as five times in succession in a volunteer (man, age 42). The coefficient of variation of each cross peak was calculated.

### Data processing

Raw L-COSY data were transferred to MATLAB [18] for signal combination from multiple elements followed by row concatenation into a two-dimensional matrix. Commercial two-dimensional spectral processing software (Felix-2007, Accelrys, San Diego, CA, USA) was used for observer-independent spectral processing and analysis. The processing parameters used were: F2 domain (skewed sine-squared window, 2,048 points, magnitude mode), F1 domain (sine-squared window, linear prediction to 96 points, zero-filling to 512 points, magnitude mode). The effect of altering time domains and window functions in two-dimensional COSY has been documented elsewhere [19]. Residual water was removed by using a Felix built-in Gaussian shaped convolution-based method. The total creatine methyl diagonal resonance at 3.02 ppm was used as an internal chemical shift reference in F1 and F2. All ‘cross’ or off-diagonal peaks are denoted with F2 – F1 in ppm units. When preparing the spectra for figures, each spectrum was calibrated by setting the lysine cross peak (at 3.00– 1.67 ppm) and specifying a constant ‘number of contour levels’ (set to 28), as well as a constant ‘level multiplier’ (defined as the difference between values of consecutive contour, set to 1.05). The volumes of cross peaks or diagonal resonances were evaluated using Felix software described above, and care was taken to ensure that the interrogated volume was the same in all L-COSY spectra. To further avoid operator bias, this was done by creating a fixed template to analyze the L-COSY data. All identifiable peaks according to Govindaraju [20], Lean [21] or Ramadan [11] were reported and peaks were measured and normalized to the creatine diagonal peak volume at 3.02 ppm for comparable results across all scans. The post processing that was ultimately chosen for the study was the same as for the glioma study [11].

### Statistical analysis

Wilcoxon ranked sum tests were performed to determine statistical significance. Bonferroni correction was not used due to the small sample size, the large number of independent variables and the pilot nature of this study. A significance level of 0.05 was utilized to compare data from the RBT cohort and healthy controls. Altman's Nomogram [22], determined by the size of the differences was employed to determine the minimum sample size for *P* ≤0.05 at a level of 90% significance for future studies.

## Results

### Variation of the L-COSY method

The *in vivo* coefficients of variation are summarized in Table 2 where the internal reference creatine had a variation of 1% while the other metabolites were 10% or less. In the phantom solutions, a variation of 5% or less was obtained.

**Table 2:** **Reproducibility of the L-COSY method: single healthy volunteer over five separate scans**

**Molecule**	**Coupling**	**F2–F1 (ppm)**	**Variation (%)**
Cr	N(C**H3**)	3.02–3.02	1
Glx-1	-C**H2**- C**H**(N**H**3+)	2.09–3.75	4
Glx-3		2.07–3.73	4
Glx-4		2.14–3.74	5
Glx-6		2.45–2.01	6
Glx-7		2.31–2.28	6
tCho	(CH3)3-N + −CH2-CH2-OH	4.05–4.05	2
Fucose region and Thr	**-CH(**OH**)-CH3** CH3-CH	(F2: 4.0–4.5, F1: 1.1–1.7)	10
Phe	-C**H**-(aromatic)-	7.33–7.33	7
NAA	-C**H3**	2.00–2.00	2
Cho	(CH3)3-N + −C**H**2-C**H**2-OH	3.51–4.05	3
	(**CH3**)3-N + −CH2-CH2-OH	3.20–3.20	5
mI	-C**H**(OH)-C**H**(OH)	3.63–3.29	5
		3.55–3.55	3

Abbreviations: Cho, choline; Cr, creatine; Glx 1-7, crosspeaks of glutamate + glutamine; L-COSY, localized correlated spectroscopy; mI, myo-inositol; NAA, N-acetyl aspartate; Phe, phenylalanine; ppm, parts per million; Thr, threonine. In bold are the proton groups that give rise to the crosspeak chemical shifts indicated in the third column.

The mean cross peak area of lysine (Lys, at 3.00–1.67 ppm) referenced to creatine was found to be the same in both RBT subjects and controls. Therefore, Lys was used to set the same contour levels across all data sets for presentation.

### Healthy versus repetitive brain trauma

**Figure 1:**
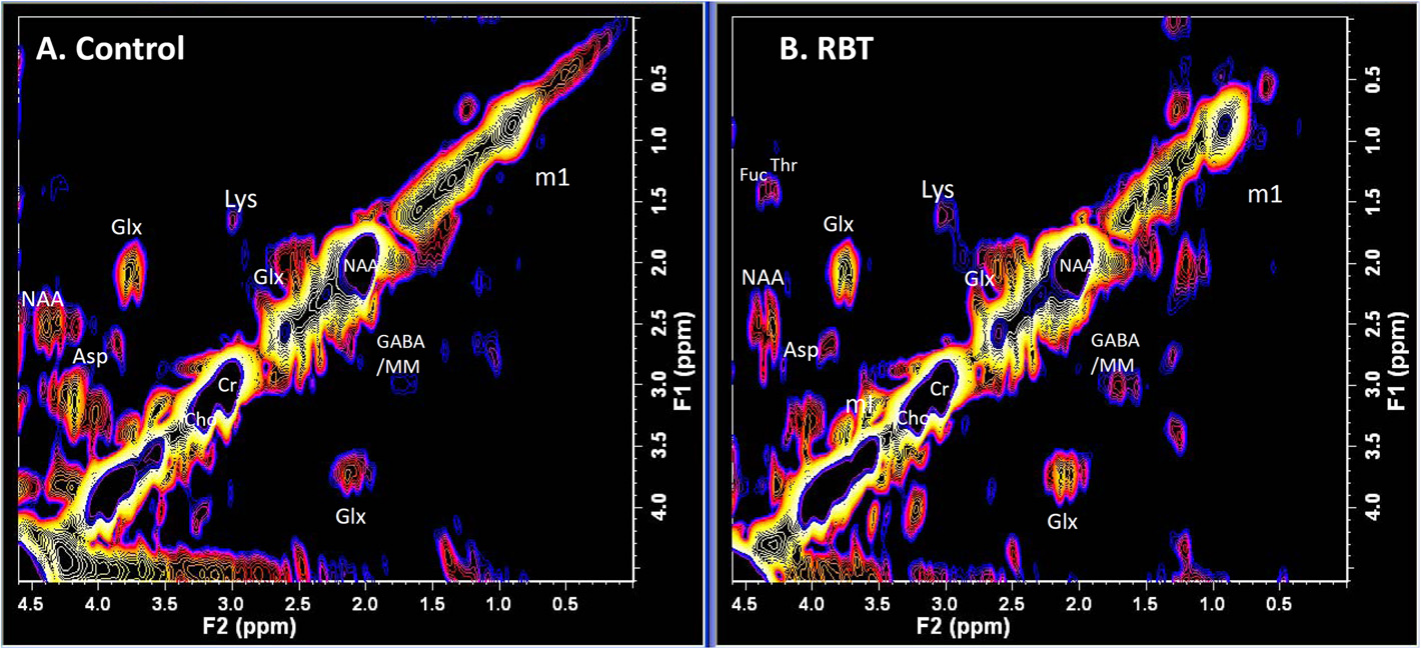
**L-COSY spectra. A)** Age-matched healthy control; **B)** Athlete with history of RBT. Spectroscopy was performed at 3 T using a 32 channel head coil and voxel size of 3 × 3 × 3 cm^3^ in the PCG; increment size 0.8 ms; 64 increments with 8 averages resulting in an acquisition time of 12.8 minutes; acquired vector 1,024 points; acquisition time 512 ms; spectral width in F2 2,000 Hz and spectral width in F1 1,250 Hz. Assigned neurochemicals are according to Ramadan *et al*. [11] and Lean [21]. For presentation, the spectra were calibrated to the lysine cross peak at 3.00–1.67 ppm. Abbreviations: N-acetylaspartate (NAA), choline (Cho); creatine (Cr); glutamate (Glu) and glutamine together (Glx); aspartate (Asp); myoinsitol (mI); lysine (Lys); threonine (Thr), gamma-aminobutyric acid (GABA). L-COSY, localized correlated spectroscopy; PCG, posterior cingulate gyrus; RBT, repetitive brain trauma.

Importantly, none of the athletes recorded structural MRI abnormalities using conventional imaging metrics. A typical L-COSY spectrum from a healthy male with no history of brain trauma is shown in Figure 1A. This is compared to L-COSY data recorded from a professional male athlete of similar age and weight, with history of RBT, and with cognitive symptoms in Figure 1B. These representative spectra demonstrate well-resolved cross and diagonal peaks including amino acids: alanine, aspartate, isoleucine, leucine, lysine, taurine, threonine (Thr), phenylalanine (Phe); membrane phospholipids: glycerophosphorylcholine, phosphocholine, phosphoethanolamine; neurotransmitters: γ-amino-butyric acid (GABA), glutamate/glutamine (Glx); major metabolites: N-acetyl aspartate (NAA), choline (Cho), creatine (Cr), myo-inositol (mI), glutathione (GSH), taurine, lactate, multiple lipid resonances, and macromolecules.

**Figure 2:**
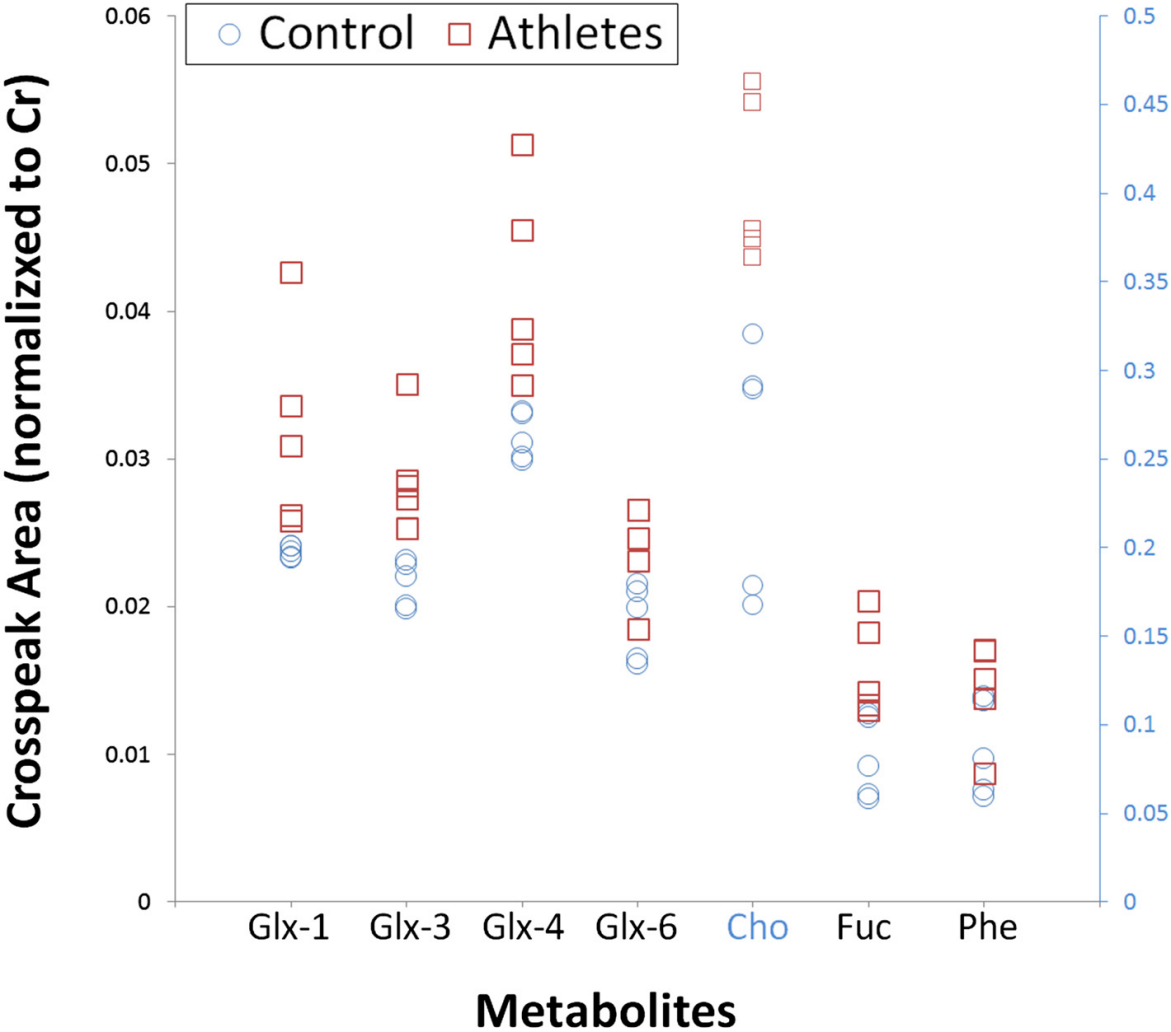
**Scatterplot of significantly different (** ***P*** **≤0.05) metabolites.** The normalized cross peak areas, as measured in the posterior cingulate gyrus using L-COSY, of the different resonances of glutamate-glutamine (Glx-1,3,4,6), choline (Cho), fucosylated glycans and threonine and phenylalanine (Phe) are shown for RBT subjects in red boxes and age-matched controls in blue circles. For clarity, choline is plotted on a secondary y-axis (right) due to the difference in the values which is highlighted in blue. L-COSY, localized correlated spectroscopy; RBT, repetitive brain trauma.

Large and significant differences in neurochemical levels were recorded when comparing age-matched RBT subjects to controls using non-parametric tests (*P* ≤0.05). Summarized in Table 3 and Figure 2 (scatterplot) are those cross peaks which are significantly different. Additional metabolites of interest such as NAA and mI (both have been shown to change in other traumatic brain injury (TBI) studies [23,24]) where changes were small or not statistically significant in this pilot series are also shown (Table 4).

**Table 3:** **Statistically significant differences between healthy and RBT subjects**

**Molecule**	**Coupling**	**F2–F1 (ppm)**	**Control mean volume/Cr**	**RBT mean volume/Cr**	**% Diff between mean and RBT**	**Effect size**
Glx-1	-CH2- CH(NH3+)	2.09–3.75	0.112 ± 0.009	0.147 ± 0.017	31 ± 18	3.11
Glx-3		2.07–3.73	0.022 ± 0.002	0.029 ± 0.004	32 ± 21	2.70
Glx-4		2.14–3.74	0.031 ± 0.002	0.042 ± 0.007	35 ± 24	2.88
Glx-6		2.45–2.01	0.018 ± 0.001	0.041 ± 0.011	77 ± 6	6.28
tCho	(CH3)3-N + −CH2-CH2-OH	4.05–4.05	0.250 ± 0.071	0.413 ± 0.051	65 ± 36	3.54
Fucose region and Thr	**-CH(**OH**)-CH3** CH3-CH	(F2: 4.0–4.5, F1: 1.1–1.7)	0.022 ± 0.007	0.036 + 0.011	64 ± 61	2.64
Phe	-CH-(aromatic)-	7.33–7.33	0.024 ± 0.005	0.035 ± 0.008	46 ± 40	2.08

Cross peak volumes were calculated relative to creatine. % Diff is the difference of the control and RBT means divided by the control mean ± the standard deviation. In bold are the proton groups that give rise to the crosspeak chemical shifts indicated in the third column.

Abbreviations: Glx, crosspeaks of glutamate + glutamine; Phe, phenylalanine; RBT, repetitive brain trauma; tCho, total choline; Thr, threonine.

**Table 4:** **Additional metabolites of interest**

**Molecule**	**Coupling**	**F2–F1 (ppm)**	**Control mean volume/Cr**	**RBT mean volume/Cr**	**% Diff between mean and RBT**	**Effect size**
NAA	-C**H3**	2.00–2.00	1.215 ± 0.101	1.294 ± 0.070	7 ± 10	0.95
tCho	(CH3)3-N + −C**H**2-C**H**2-OH	3.51-4.05	0.018 ± 0.002	0.020 ± 0.016	11 ± 81	0.23
	(**CH3**)3-N + −CH2-CH2-OH	3.20-3.20	0.726 ± 0.009	0.774 ± 0.052	7 ± 7	1.63
mI	-C**H**(OH)-C**H**(OH)	3.63–3.29	0.029 ± 0.013	0.032 ± 0.011	10 ± 61	0.26
		3.55–3.55	0.342 ± 0.013	0.354 ± 0.029	4 ± 10	0.58

Data are presented as described in Table 3, that is, relative to creatine. In all cases, the differences between control and RBT were not statistically significant.

Abbreviations: mI, myo-inositol; NAA, N-acetyl aspartate; RBT, repetitive brain trauma; tCho, total choline. In bold are the proton groups that give rise to the crosspeak chemical shifts indicated in the third column.

The RBT cohort recorded an increase of 31%, 32% and 35% in the glutamine/glutamate cross peaks (2.09–3.75 ppm, 2.07–3.73 ppm and 2.14–3.74 ppm, respectively) (Table 3) when compared to controls. Figure 2 clearly demonstrates that for most of the Glx resonances there is no overlap between the RBT and control cohorts. It remains contentious as to whether glutamate and glutamine resonances are separable; thus, it cannot yet be ascertained if the increase is in glutamine or glutamate or both.

The methylene group of choline at 4.05–4.05 ppm was 65% higher in the RBT cohort. The scatterplot (Figure 2) shows that there is no overlap between the cross peak values measured in RBT subjects and controls. It is interesting to note that while the mean peak volumes of the choline resonances at 3.20–3.20 ppm and 3.51–4.05 ppm were greater, they were not statistically significant.

L-COSY also uniquely identifies additional resonances that cannot be characterized by one-dimensional MRS methods. For example, the cross peaks in the F2: 4.0–4.5, F1: 1.1–1.7 region, show a higher cross peak volume in molecules assigned to covalently linked terminal fucose molecules and threonine [21] of 60% in the RBT group [21]. The spectral region (F2: 4.0–4.5, F1: 1.1–1.7) contains threonine, fucose and, sometimes, lactate cross peaks. Originally assigned in the two-dimensional COSY from cultured cells [21,25] and more recently *in vivo* in the brain [11], these fucose cross peaks are also seen in the brain spectra reported by Velan *et al*. [14], but assigned to Thr/lactate alone. The assignment of bound fucose in the two-dimensional MR spectra was previously made using *in vitro* models by treating the cultured cells with fucosyl transferase and observing the bound cross peaks disappear and free α fucose at 1.22–4.21 ppm and free *β* fucose at 1.26–3.81 ppm appear [25]. It is the unique conformation of each of these terminal fucosylated species on the oligosaccharide chain on the cell membrane that generates the different chemical shifts for each fucose. Replicating this in a phantom is, thus, not possible [26].

**Figure 3:**
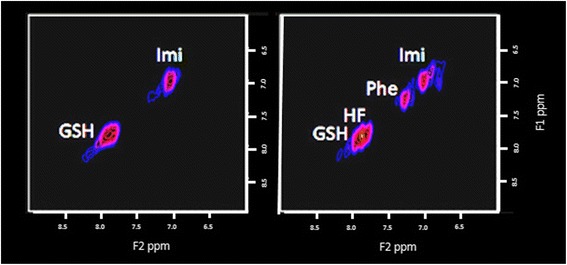
**An expansion of the aromatic region (F2: 6.00–9.00 ppm; F1: 6.00–9.00 ppm).** Resonances from phenylalanine (Phe; 7.33–7.33); imidazole from histamine and homocarnosine (Imi; 7.07–7.07); and high-frequency peak from NAA amine group and imidazole from histamine and histidine (HF; 7.82–7.82) are on the diagonal. The data are again expanded from Figure 1. On the left are the results from the healthy control and on the right from the athletes with RBT, in which phenylalanine cross peaks are well visualized. There is a mean statistically significant greater phenylalanine by 46% in the RBT group as a whole. NAA, N-acetyl aspartate; RBT, repetitive brain trauma.

An expansion of the aromatic region (F2: 6.00–9.00 ppm; F1: 6.00–9.00 ppm) where resonances [20] from phenylalanine (Phe; 7.33–7.33 ppm); imidazole from histamine and homocarnosine (Imi; 7.07–7.07 ppm); and high-frequency peak from NAA amine group and imidazole from histamine and histidine (HF; 7.82–7.82 ppm) on the diagonal are shown in Figure 3 as expanded from Figure 1. Phenylalanine was 46% higher in RBT subjects compared with controls and statistically significant.

Based on the literature, two other molecules expected to increase with RBT were NAA and myo-inositol (Table 4). They were increased by 7% and 10%, respectively; however, these changes were relatively small and they did not reach statistical significance [22]. This is of particular interest given that these metabolites have been shown to be significantly altered in other studies of head injury but do not appear to be different in this limited cohort.

## Discussion

### Neurochemical changes with chronic repetitive brain trauma

This study demonstrates that the L-COSY method documents neurochemical differences in former professional athletes with a history of RBT compared to healthy age-matched controls with no history of head injury. Changes in cerebral metabolites after severe TBI, in both children and adults, have shown strong correlations with clinical grade and patient outcome in previous studies [16,27,28]. The changes expected from previous one-dimensional MRS of sports-related TBI’s [9,10,29] included changes in NAA, choline and glutamine in acute and sub-acute stages of injury. However, this is the first study to examine the chronic changes in athletes with a history of RBT and the first to apply two-dimensional L-COSY methodology. An important outcome from this study is the ability to monitor a larger range of molecules with greater accuracy. For example, large increases in glutamine/glutamate, choline and phenylalanine are evident and molecules such as the fucosylated glycans are now available for inspection in the brain *in vivo*. While the changes observed in this cohort are not necessarily representative of the heterogeneous population of athletes with RBT, these preliminary results may provide new insights into the biochemical pathways being altered as a function of RBT.

The composite glutamate/glutamine was over 30% higher in the athletes with RBT. Glutamate is a major excitatory neurotransmitter and neuronal biomarker, the increase in which is known to be predictive of poor outcome in severe TBI [30]. Glutamine, the second metabolite contributing to the region designated Glx, is a predominantly glial metabolite and may, therefore, provide information relevant to the gliosis or neuroinflammation identified in other studies of Alzheimer disease (AD) [31] and hepatitis [32]. TBI alters the brain homeostasis resulting in the activation of microglia via proinflammatory cytokines [33] and in mouse models of CTE, repetitive brain injury resulted in augmentation of tau pathology as well as glial activation [34]. Recently, resting and activated glial density was shown to be increased in subjects with CTE when compared with controls, and those with CTE and motor neuron disease showed even greater glial density [35]. Another link to CTE can be found in the clinical features of CTE. Recent work has shown, through next-of-kin interviews of 36 athletes all with neuropathologically confirmed CTE, a distinct initial clinical presentation at a younger age of behavioral and mood disturbances and initial presentation at an older age that involved cognitive impairment [36]. Several studies have shown that changes in glutamate occur in depression and other mood disorders [37,38].

Choline was 65% higher in RBT subjects. Importantly, there was no overlap between the choline values of each group, thereby demonstrating a distinct difference between the RBT and control groups. Previous studies have demonstrated that increased total choline which includes several related membrane metabolites (phosphocholine, glycerophosphocholine, phosphoethanolamine, glycerophosphoethanolamine, and so on) is reflective of brain tissue damage or diffuse axonal injury in TBI [39]. It is unclear at this time if the unambiguous choline measured by COSY is part of the same mechanism but the large difference in this small cohort bears further study.

The cross peaks assigned to fucosylated glycans at (F1 0.90–1.70; F24.0–4.50) increased by 60% in subjects with RBT. The levels of fucosylated epitopes, for example, sialyl Lewis x, have been shown to increase during the early events of inflammation [40-43]. Others have shown that fucosylation of glycoproteins increases in inflammation which may be related to the inflammation shown in chronic brain injury by positron emission tomography (PET) studies [44]. In the healthy brain fucose-α(1–2)-galactose (Fucα(1–2)Gal] sugars have been implicated in the molecular mechanisms that underlie neuronal development, learning and memory [26]).

Phenylalanine, increased by 46% in the brains of the RBT cohort, can be converted into tyrosine - another of the DNA-encoded amino acids. Tyrosine, in turn, is converted into 3,4-dihydroxyphenylalanine (Dopa), which is further converted into dopamine, noradrenalin and adrenaline [45]. This elevation may well be the result of interference with a critical part of the noradrenergic and adrenergic neurotransmitter pathways after RBT. Phenylalanine in increased quantities also interferes with the production of serotonin, an imbalance of which is thought to influence mood in a way that leads to depression [46].

Finally, mI was not found to be significantly different in RBT subjects. Previous studies have shown that brain trauma is a risk factor for dementia [47,48] which has led some to believe that the underlying cause for dementia following TBI may be CTE and not AD in itself [49,50]. Although CTE is neuropathologically distinct from AD [4], the same study also showed that 11% of the American football players had co-morbid AD. Furthermore, the differential diagnosis of these two diseases in life is difficult if not possible based solely on clinical presentation. Just as in AD, initial symptoms of CTE can include impaired episodic memory and in late stage CTE dementia can be clinically mistaken for AD [2]. As a result, the relationship between TBI and neurodegenerative disease, such as AD, will remain unclear until a two-dimensional L-COSY study is undertaken on AD and a larger RBT cohort.

### Study limitations and future studies

A major limitation to this study is the small sample size. The goal of this pilot investigation was to establish initial data on the utility of L-COSY MRS in distinguishing individuals with a history of RBT from healthy controls. It is not possible to determine if the changes observed in this small sample of former athletes with histories of RBT are indicative of the progressive neurodegenerative disease CTE or are a reflection of the residual, chronic effects of the initial brain injuries. Furthermore, head injury is heterogeneous and, thus, a larger sample may have a greater variability that is not reflected in this small cohort. Additional studies are planned to focus on this and other important issues with the goal of utilizing L-COSY MRS as a biomarker for CTE.

The capacity to measure changes in this larger number of neurochemicals at one time, with high accuracy, and on a personalized basis, is now possible using the L-COSY technique. Such an approach could alter long term care of the patients with RBT and other neurological diseases. Much interest lies in clarification as to whether the data presented here are the result of persistent, chronic post-concussion syndrome and/or the neurodegenerative disease identified post mortem as CTE. This question will not be answered quickly as individuals need to be followed over time. However, of more immediate use is the L-COSY method to assist with a multi-modal biomarker diagnostic approach to CTE, leading to the ability to examine risk factors and prevalence of this neurodegenerative disease, as well as to understand better the prevention and treatment of RBT.

## Conclusions

This study demonstrates that two-dimensional L-COSY can document neurochemical changes in former professional athletes who have experienced RBT and symptoms including headaches, memory loss, confusion, impaired judgment, impulse control problems, aggression and depression. Large and significant increases in the neurochemicals glutamate/glutamine and choline are recorded as expected from earlier studies but phenylalanine and fucosylated molecules have not been recorded previously in RBT or mTBI. The literature links these four neurochemicals to glutamatergic neurotoxicity, diffuse axonal injury, neurotransmitter dysfunction and neuro inflammation. These results need to be confirmed by future studies with a larger cohort that may provide the basis for developing in life diagnosis of CTE. For smaller differences recorded, the appropriate group sizes were calculated to guide future study sizes.

## Abbreviations

AD: Alzheimer’s disease
Asp: aspartate
Aβ: beta-amyloid
Cho: choline
COSY: correlated spectroscopy
Cr: creatine
CTE: chronic traumatic encephalopathy
CV: coefficient of variation
Dopa: 3,4-dihydroxyphenylalanine
Fuc: fucosylated Protein
GABA: gamma-aminobutyric acid
Glu: glutamate
Glx: glutamate and glutamine
HF: high-frequency peak (from NAA amine group)
Imi: imidazole
L-COSY: localized correlated spectroscopy
Lys: lysine
mI: myoinositol
MRS: magnetic resonance spectroscopy
mTBI: mild traumatic brain injury
NAA: N-acetylaspartate
PCG: posterior cingulate gyrus
PET: positron emission tomography
RBT: repetitive brain trauma
TBI: traumatic brain injury
Thr: threonine
TR: repetition time
